# Agreement between MRI and pathologic breast tumor size after neoadjuvant chemotherapy, and comparison with alternative tests: individual patient data meta-analysis

**DOI:** 10.1186/s12885-015-1664-4

**Published:** 2015-10-08

**Authors:** Michael L. Marinovich, Petra Macaskill, Les Irwig, Francesco Sardanelli, Eleftherios Mamounas, Gunter von Minckwitz, Valentina Guarneri, Savannah C. Partridge, Frances C. Wright, Jae Hyuck Choi, Madhumita Bhattacharyya, Laura Martincich, Eren Yeh, Viviana Londero, Nehmat Houssami

**Affiliations:** 1 Screening and Test Evaluation Program (STEP), Sydney School of Public Health, The University of Sydney, A27, Edward Ford Building, Sydney, NSW 2006 Australia; 2 Dipartimento di Scienze Biomediche per la Salute, Università degli Studi di Milano, Unità di Radiologia, IRCCS Policlinico San Donato, Piazza E. Malan 2, San Donato Milanese, Milano Italy; 3 MD Anderson Cancer Center Orlando, 1400 South Orange Avenue, MP 700, Orlando, FL 32806 USA; 4 German Breast Group & Universitäts-Frauenklinik Frankfurt, Martin-Behaim-Str. 12, 63263 Neu-Isenburg, Germany; 5 University of Padova, Division of Medical Oncology 2, Istituto Oncologico Veneto IRCCS, Padova, Italy; 6 Department of Radiology, University of Washington, 825 Eastlake Ave E, G3-200, Seattle, WA 98109-1023 USA; 7 Division of General Surgery, Sunnybrook Health Sciences Centre, 2075 Bayview Avenue, Toronto, ON M4C 5T2 Canada; 8 School of Medicine, Jeju National University Hospital, Aran 13gil 15(ara-1 dong), Jeju-si, Jeju-do South Korea; 9 Berkshire Cancer Centre, Royal Berkshire NHS Foundation Trust, London Road, Reading, RG1 5AN UK; 10 Direzione Radiodiagnostica, Fondazione del Piemonte per l’Oncologia-IRCCS, Str. Prov.142, Candiolo, Torino Italy; 11 Department of Radiology, Brigham and Women’s Hospital, 75 Francis St, Boston, MA 02115 USA; 12 Institute of Radiology, University of Udine, p.le S.M. della Misericordia, 15, 33100 Udine, Italy

**Keywords:** Breast cancer, Neoadjuvant chemotherapy, Magnetic resonance imaging, Tumor response, Monitoring

## Abstract

**Background:**

Magnetic resonance imaging (MRI) may guide breast cancer surgery by measuring residual tumor size post-neoadjuvant chemotherapy (NAC). Accurate measurement may avoid overly radical surgery or reduce the need for repeat surgery. This individual patient data (IPD) meta-analysis examines MRI’s agreement with pathology in measuring the longest tumor diameter and compares MRI with alternative tests.

**Methods:**

A systematic review of MEDLINE, EMBASE, PREMEDLINE, Database of Abstracts of Reviews of Effects, Heath Technology Assessment, and Cochrane databases identified eligible studies. Primary study authors supplied IPD in a template format constructed *a priori*. Mean differences (MDs) between tests and pathology (i.e. systematic bias) were calculated and pooled by the inverse variance method; limits of agreement (LOA) were estimated. Test measurements of 0.0 cm in the presence of pathologic residual tumor, and measurements >0.0 cm despite pathologic complete response (pCR) were described for MRI and alternative tests.

**Results:**

Eight studies contributed IPD (N = 300). The pooled MD for MRI was 0.0 cm (LOA: +/−3.8 cm). Ultrasound underestimated pathologic size (MD: −0.3 cm) relative to MRI (MD: 0.1 cm), with comparable LOA. MDs were similar for MRI (0.1 cm) and mammography (0.0 cm), with wider LOA for mammography. Clinical examination underestimated size (MD: −0.8 cm) relative to MRI (MD: 0.0 cm), with wider LOA. Tumors “missed” by MRI typically measured 2.0 cm or less at pathology; tumors >2.0 cm were more commonly “missed” by clinical examination (9.3 %). MRI measurements >5.0 cm occurred in 5.3 % of patients with pCR, but were more frequent for mammography (46.2 %).

**Conclusions:**

There was no systematic bias in MRI tumor measurement, but LOA are large enough to be clinically important. MRI’s performance was generally superior to ultrasound, mammography, and clinical examination, and it may be considered the most appropriate test in this setting. Test combinations should be explored in future studies.

**Electronic supplementary material:**

The online version of this article (doi:10.1186/s12885-015-1664-4) contains supplementary material, which is available to authorized users.

## Background

Magnetic resonance imaging (MRI) has been proposed to have a role in guiding breast cancer surgery by measuring the size of residual tumor after neoadjuvant chemotherapy (NAC), and has been shown to have high sensitivity for detecting residual disease [1]. Given that guidelines recommend assessment of the largest tumor diameter [2], estimation of the largest diameter by MRI may guide decisions about whether subsequent mastectomy or breast conserving surgery (BCS) should be attempted, as well as assist in planning resection to achieve clear margins in BCS. Underestimation of tumor size may therefore lead to involved surgical margins and repeat surgery; overestimation may lead to overly radical surgery (including mastectomy when BCS may have been possible), and poorer cosmetic and psychosocial outcomes [3].

Tumor size measurement is subject to potential errors, and both tumor characteristics and imaging limitations may differentially affect the measurement accuracy of tests used for this purpose. MRI may over- or underestimate tumor size due to artefacts such as partial volume effects [4] or disruptions to signal intensity from marker placement [5]. Tumors may not be well visualised by mammography in patients with dense breasts [6] or multifocal cancer [7]. Ultrasound (US) measurements may be compromised by unclear margins [8], acoustic shadowing [9] or limitations in the field of view [10]. Imaging modalities also differ in their ability to visualise ductal carcinoma *in situ* (DCIS) [11]. The inherent pliability of breast tissue also means that tumor dimensions may vary depending on patient positioning [12]; therefore, differences in measurements undertaken in upright (mammography), supine (US) and prone positions (MRI) may arise. Furthermore, the effects of NAC may introduce greater bias in residual tumor measurement relative to the preoperative setting: reactive inflammation, fibrosis or necrosis may be difficult to distinguish from residual tumor [13], and measurement errors may be additive when tumors regress as multiple, scattered deposits [2].

While many studies have sought to assess the relative ability of MRI and other tests to estimate tumor size after NAC, conclusions have been hampered by small sample sizes and inadequate statistical methods. A previous study-level meta-analysis demonstrated that misleading conclusions about the accuracy of MRI may result from inappropriate analytic methods that do not measure agreement between clinical measures (e.g. Pearson or Spearman correlation coefficients) [14]. However, that meta-analysis was limited in its ability to estimate the agreement between MRI and pathologic measurements, and to compare MRI with alternative tests, due to numerous shortcomings in the available data. For example, inconsistencies in measurement between studies, such as the inclusion or exclusion of residual ductal carcinoma in situ (DCIS) in pathologic tumour measurements, may differentially affect the measurement accuracy of MRI and other tests, and also limit the clinical applicability of pooled estimates. Comparison of MRI and other tests was also hampered by the tests being reported for different (or, at best, overlapping) patient groups, for which test performance may vary. Furthermore, a fundamental limitation was that assessing the validity of assumptions underlying the recommended statistical methods (mean differences and limits of agreement [15]) was often not possible due to inadequate reporting.

To address those limitations, we investigated agreement between MRI-measured and pathologic tumor size after NAC in an individual patient data (IPD) meta-analysis of a large number of breast cancer patients, using appropriate methods for evaluating the agreement between measurements [15]. Key differences between this and the previous study-level meta-analysis are summarised in Additional file 1: Appendix 1. The IPD methodology allowed us to standardise tumor measurements to include invasive cancer only, explore agreement only when residual tumor is truly present, and describe MRI measurement errors in detail. In addition, our study extended previous work by exploring agreement by characteristics that have been suggested to contribute to inaccurate measurement (NAC agents and HER2 status) [16, 17], and examining MRI’s agreement compared with and in addition to alternative tests (US, mammography, clinical examination) when the tests were conducted in the same patients [18].

## Methods

### Identification of studies

A systematic literature search up to February 2011 was undertaken to identify studies of MRI for measuring residual tumor after NAC. MEDLINE and EMBASE were searched via EMBASE.com; PREMEDLINE, Database of Abstracts of Reviews of Effects, Heath Technology Assessment, and Cochrane databases were searched via Ovid. Search terms linked MRI with breast cancer and response to NAC. Keywords and medical subject headings included ‘breast cancer’, ‘nuclear magnetic resonance imaging’, ‘MRI’, ‘neoadjuvant’, and ‘response’. The full search strategy has been reported previously [1, 19]. Reference lists were also searched and content experts consulted to identify additional studies.

### Review of studies and eligibility criteria

Abstracts were screened for eligibility by one author (MLM); a sample of 10 % was assessed independently (NH) to ensure consistent application of eligibility criteria. There were no changes to eligibility criteria or coding schemes based on the independent assessment. Eligible studies enrolled ≥15 patients with newly diagnosed breast cancer undergoing NAC, with MRI *and at least one other test* (US, mammography, clinical examination) *after* NAC to assess residual tumor size (longest diameter) prior to surgery.

Potentially eligible citations were reviewed in full (MLM or NH). The screening and inclusion process is summarised in Additional file 1: Appendix 2.

### Individual patient data

A research protocol and database template were drafted *a priori*, specifying the study rationale and objectives, IPD requirements, and planned statistical analyses (Additional file 1: Appendix 3). Those documents were forwarded to the authors of eligible studies with an invitation to participate in the IPD meta-analysis, with email follow-up if no response was received.

For each participating study, data irregularities were discussed with the authors. Non-numeric tumor measurements were treated as missing data. Observations with missing pathologic measurements were excluded. Pathologic measurements considered residual invasive components only; therefore, the definition of pathologic complete response (pCR) was standardised across studies as the absence of residual invasive cancer, with or without the presence of DCIS (i.e. a pathologic measurement of 0.0 cm) [20].

### Statistical analysis

For individual studies, Bland-Altman scatterplots of the differences between measurements by the relevant tests and pathology (vertical axis) and their mean (horizontal axis) were constructed. Plots were examined to assess whether the differences were normally distributed and independent from the underlying size of the measurements [15]. Scatterplots of log-transformed measurements were also constructed to assess whether underlying relationships were improved. Preliminary mixed linear models (PROC MIXED in SAS) of the difference between measurements by their mean, and pathologic size by MRI size, were unstable and are not reported.

For patients with residual tumor at pathology, measurement biases were estimated as the *absolute* mean differences (MDs) between MRI, comparator tests and pathology; the associated 95 % limits of agreement (LOA) were also calculated for each study [15]. *Relative* MDs were derived by exponentiation of the difference of log-transformed measurements. MDs were pooled by the inverse variance method using RevMan 5.2. A fixed effect was assumed unless statistically significant heterogeneity was present, as assessed by the Cochrane Q statistic. The extent of heterogeneity was quantified by the I^2^ statistic [21]. To estimate the 95 % LOA for a pooled MD, a pooled variance was computed under the assumption that the variance of the differences was equal across studies. The pooled variance was calculated as the weighted average of these within-study variances, weighted by the corresponding degrees of freedom for each study (i.e. an extension of the approach used for a two sample *t*-test [22]).

In addition, test measurements of 0.0 cm in the presence of pathologic residual tumor, and measurements >0.0 cm despite pCR were described for MRI and comparator tests. Exact 95 % confidence intervals for proportions were computed (SAS version 9.2). Paired differences between tests were tested with McNemar’s test. Differences in characteristics between patients with and without tumor measurements by comparator tests were compared with independent samples t-tests for continuous variables and with chi-squared or Fisher’s exact tests for categorical variables.

All tests of statistical significance were two-sided. Except for tests of heterogeneity (*p* < 0.10), the level chosen for statistical significance was *p* < 0.05; *p* ≤ 0.10 was considered to represent weak evidence of a difference [23].

## Results

### Study characteristics

A total of 2108 citations were identified. Twenty-four studies (1228 patients) were eligible for inclusion [13, 24–46]; eight of those contributed IPD to this analysis (300 patients) [13, 24, 25, 29, 34, 38, 44, 46] (Additional file 1: Appendix 2). Agreement between residual tumor size by tests and pathology was compared for MRI and US in five studies [13, 29, 34, 38, 46]; MRI and mammography in four studies [13, 24, 34, 38]; and MRI and clinical examination in three studies [13, 24, 25]. For one study [44], MRI and pathologic measurements were provided but data for alternative tests were unavailable.

Characteristics of the included studies are presented in Table 1. Included studies were generally representative of the broader population of studies reported previously, based qualitative comparison of aggregate descriptive characteristics [14]. However, patients in this analysis were more likely to have had T3 tumors or stage III disease; were more commonly treated with anthracycline-taxane-based NAC; and had a shorter time between MRI and surgery.

**Table 1:** Summary of cohort, tumour, treatment and reference standard characteristics of studies included in the individual patient data analysis

		Study level estimates		
Variable	Patients (%)	Median	IQR	Range
*Cohort characteristics*				
N patients with MRI (8 studies)	300 (NA)	36	28 – 50	13 – 59
Recruitment mid-point (year) (4 studies)	144 (NA)	2003	2001 – 2005	2001 – 2006
Age, mean or median (years) (8 studies)	300 (NA)	47	46 – 48	43 – 49
Menopausal status (%)^a^ (2 studies)				
Pre	51 (72.9)	64.6	60.4 – 68.8	60.4 – 68.8
Peri/post	19 (27.1)	25.0	18.8 – 31.2	18.8 – 31.2
*Tumour characteristics*				
Clinical size, mean or median (cm)^a^ (4 studies)	136 (NA)	4.6	4.2 – 6.6	4.0 – 8.2
T stage (%)^a^ (4 studies)				
T1	5 (2.9)	2.9	1.0 – 5.0	0.0 – 6.2
T2	62 (35.8)	43.0	23.9 – 50.3	6.2 – 56.2
T3	78 (45.1)	43.6	38.3 – 49.1	37.5 – 50.0
T4	28 (16.2)	9.8	0.0 – 30.6	0.0 – 41.7
Stage (%)^a^ (6 studies)				
I	1 (0.5)	0.0	0.0 – 0.0	0.0 – 3.1
II	131 (59.0)	66.1	45.0 – 78.0	27.1 – 86.7
III	83 (37.4)	32.3	22.0 – 37.5	0.0 – 72.9
IV	7 (3.2)	0.0	0.0 – 0.0	0.0 – 17.5
Histology (%)^a^ (6 studies)				
IDC	191 (84.1)	86.2	74.2 – 87.8	68.8 – 90.0
ILC or IDC/ILC	19 (8.4)	9.8	5.1 – 10.0	4.9 – 18.8
Other	17 (7.5)	7.9	4.0 – 12.5	0.0 – 16.1
Nodal status (%)^a^ (4 studies)				
Positive	109 (72.2)	71.0	62.5 – 80.6	56.2 – 87.8
Negative	42 (27.8)	29.0	19.4 – 37.5	12.2 – 43.8
ER (%)^a^ (5 studies)				
Positive	113 (60.1)	62.5	60.0 – 64.4	45.0 – 69.2
Negative	73 (38.8)	37.5	32.2 – 40.0	15.4 – 55.0
Unknown or NR	2 (1.1)	0.0	0.0 – 0.0	0.0 – 3.4
PR (%)^a^ (4 studies)				
Positive	71 (44.9)	41.2	32.9 – 49.8	30.8 – 52.1
Negative	84 (53.2)	51.5	47.5 – 59.4	48.5 – 65.0
Unknown or NR	3 (1.9)	1.0	0.0 – 2.7	0.0 – 3.4
HER2 (%) (3 studies)				
Positive	42 (28.8)	29.2	22.5 – 33.9	22.5 – 33.9
Negative	97 (66.4)	62.5	61.0 – 77.5	61.0 – 77.5
Unknown or NR	7 (4.8)	5.1	0.0 – 8.3	0.0 – 8.3
*Treatment*				
NAC regimen (%)^a^ (8 studies)				
Anthracycline-based	115 (38.1)	9.3	0.0 – 77.6	0.0 – 100.0
Antracycline-taxane-based	181 (59.9)	88.1	17.4 – 100.0	0.0 – 100.0
Other	6 (2.0)	0.0	0.0 – 2.5	0.0 – 100.0
Trastuzumab (%)^a^ (3 studies)				
Trastuzumab used	29 (19.6)	7.3	2.1 – 42.4	2.1 – 42.4
Trastuzumab not used	119 (80.4)	92.7	57.6 – 97.9	57.6 – 97.9
Type of surgery (%)^a^ (8 studies)				
BCS	132 (43.1)	50.3	37.6 – 55.9	6.2 – 59.4
Mastectomy	172 (56.2)	57.2	44.1 – 63.8	34.4 – 93.8
No surgery	2^b^ (0.7)	0.0	0.0 – 0.0	0.0 – 6.2
*Reference standard*				
Type of reference standard (%) (8 studies)				
Pathology	298 (99.3)	100.0	100.0 – 100.0	93.8 – 100.0
Other	2^b^ (0.7)	0.0	0.0 – 0.0	0.0 – 6.2
Time from MRI to surgery, mean or median/estimate (days) (6 studies)	228 (NA)	16	12 – 25	7 - 28
Prevalence of pCR (%) (8 studies)	300 (NA)	19.0	15.5 – 23.4	7.1 – 27.5

*BCS* breast conserving surgery, *DCIS* ductal carcinoma *in situ*, *ER* estrogen receptor, *HER2* human epidermal growth factor receptor 2, *IDC* invasive ductal carcinoma, *ILC* invasive lobular carcinoma, *IQR* inter-quartile range, *MRI* magnetic resonance imaging, *NA* not applicable, *NAC* neoadjuvant chemotherapy, *NR* not reported, *pCR* pathologic complete response, *PR* progesterone receptor

^a^Calculation of values based on total number of patients enrolled, a minority of whom may not have contributed data to this analysis

^b^Localisation biopsy showed the absence of residual tumour (i.e. pathologic measurement of 0.0 cm)

Technical characteristics of MRI are presented in Additional file 1: Appendix 4. The majority of studies used dynamic contrast-enhanced MRI (88 %) with a 1.5-T magnet (75 %). Dedicated bilateral breast coils were used in all studies reporting the coil type. All studies providing detail on contrast employed gadolinium-based materials, most commonly gadopentetate dimeglumine (62 %), at the standard dosage of 0.1 mmol/kg body weight (75 %).

Pathology from surgical excision was the reference standard for all patients in all but one study [34], where pCR was verified by localisation biopsy in two cases (0.7 % of all patients).

### MRI when residual tumor present at pathology

**Fig. 1:**
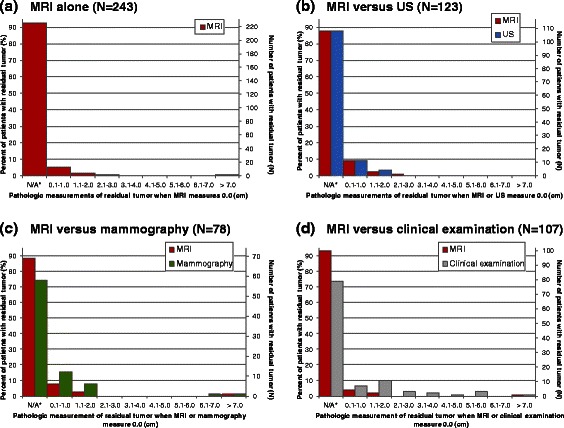
Pathologic size (cm) of tumor “missed” by MRI for: **a** all patients with residual tumor (*N* = 243); and compared with **b** US (*N* = 123), **c** mammography (*N* = 78), and **d** clinical examination (*N* = 107). MRI = magnetic resonance imaging; N/A = not applicable; US = ultrasound. *Pathology and test(s) measure > 0.0 cm (i.e. residual tumor was not “missed” by MRI or alternative tests).

Figure 1a describes the size of residual tumor present at pathology (*N* = 243) that was “missed” by MRI (i.e. MRI tumor measurements of 0.0 cm). Patients for whom MRI truly detected residual tumor (i.e. measurements > 0.0 cm) are also included in the column labelled “not applicable” (N/A). Pathologic measurements of tumors “missed” by MRI ranged between 0.1-11.0 cm (median = 0.6 cm), and measured 0.1-1.0 cm for 12 patients (4.9 %); 1.1-2.0 cm for four patients (1.6 %); 2.1-3.0 cm for one patient (0.8 %); and >7.0 cm for one patient (0.8 %).

Study-specific Bland-Altman plots, MDs and LOA between MRI and pathology are presented in Additional file 1: Appendix 5. The plots suggested a tendency in some studies for larger differences with increasing tumor size; underlying relationships were not uniformly improved by log transformation (Additional file 1: Appendix 5). Similar relationships were also apparent for US, mammography and clinical examination (Additional file 1: Appendices 6–8). Analyses of *absolute* differences between tests and pathology are reported here; analyses of *relative* (log) differences were comparable, and are presented in Additional file 1: Appendices 9–10.

Meta-analysis of MDs between MRI and pathology (Table 2; Additional file 1: Appendix 11) showed no systematic bias in MRI’s estimation of pathologic tumor size (pooled MD = 0.0 cm [95 % CI: −0.1-0.2 cm]), with no evidence of heterogeneity (I^2^ = 0 %). Scatterplots showed both over- and underestimation by MRI (Additional file 1: Appendix 5). Pooled LOA indicated that 95 % of pathologic measurements fall between +/−3.8 cm of the MRI measurement.

**Table 2:** Pooled absolute differences (cm) (fixed effect unless noted) and limits of agreement for studies and patients comparing the respective tests

	N (studies)	N (patients)	Pooled MD (cm) (95 % CI)	I^2^	LOA (cm)
All studies and patients					
MRI vs pathology	8	243	0.0 (−0.1, 0.2)	0 %	+/−3.8
Studies of MRI vs US					
MRI vs pathology	5	123	0.1 (−0.2, 0.3)	0 %	+/− 2.8
US vs pathology^a^	5	123	−0.3 (−0.6, 0.1)	69 %	+/− 2.6
MRI *and* US (mean) vs pathology	5	123	−0.1 (−0.3, 0.1)	16 %	+/− 2.3
MRI vs US^a^	5	123	0.3 (−0.1, 0.7)	81 %	NA
*MRI vs pathology (patients without US)*^*b*^	*3*	*14*	*−1.5 (−3.1, 0.1)*	*NA*	*+/− 6.0*
Studies of MRI vs mammography					
MRI vs pathology	4	78	0.1 (−0.1, 0.3)	0 %	+/− 4.1
Mammography vs pathology	4	78	0.0 (−0.3, 0.4)	39 %	+/− 5.0
MRI *and* mammography (mean) vs pathology	4	78	0.1 (−0.1, 0.4)	21 %	+/− 4.2
MRI vs mammography	4	78	0.1 (−0.2, 0.4)	0 %	NA
*MRI vs pathology (patients without mammography)*^*b*^	*3*	*25*	*0.0 (−0.7, 0.7)*	*NA*	*+/− 3.5*
Studies of MRI vs clinical examination					
MRI vs pathology	3	107	0.0 (−0.2, 0.3)	0 %	+/− 4.2
Clinical examination vs pathology^a^	3	107	−0.8 (−1.5, −0.1)*	57 %	+/− 5.1
MRI *and* clinical examination (mean) vs pathology	3	107	−0.2 (−0.5, 0.1)	9 %	+/− 4.1
MRI vs clinical examination^a^	3	107	0.9 (0.2, 1.5)*	56 %	NA
*MRI vs pathology (patients without clinical examination)*^*b*^	*2*	*3*	*NA* ^*c*^	*NA* ^*c*^	*NA* ^*c*^

*CI* confidence interval, *LOA* limits of agreement, *MD* mean difference, *MRI* magnetic resonance imaging, *NA* not applicable, *US* ultrasound

*p < 0.01

^a^Random effects

^b^Patients without comparator test combined as a single data set. Pooled meta-analysis not undertaken

^c^Not calculated due to small number of patients

#### MRI versus US

In 123 patients with pathologic residual tumor and paired measurements by MRI and US, distributions of pathologic size were comparable when either test measured 0.0 cm; tumors “missed” by each test typically measured ≤2.0 cm, with one MRI measurement in the range of 2.1-3.0 cm (Fig. 1b).

Pooled MDs showed a tendency for MRI to slightly overestimate pathologic tumor size (MD = 0.1 cm) with no evidence of heterogeneity (I^2^ = 0 %) (Table 2; Additional file 1: Appendix 11). A larger tendency for underestimation by US (MD = −0.3 cm) was observed with substantial heterogeneity (Q = 13.11, df = 4, *p* = 0.01; I^2^ = 69 %); the pooled MD did not change when a fixed or random effect(s) were assumed. Pooled differences between MRI and US showed only weak evidence of a difference between the measurements (assuming random effects, *p* = 0.10). Pooled LOA were comparable for MRI (+/−2.8 cm) and US (+/−2.6 cm) (Table 2), with both over- and underestimation observed for both tests (Additional file 1: Appendices 5–6). Combining MRI and US measurements by taking their mean resulted in slight underestimation (MD = −0.1 cm), with a small reduction in LOA compared with either test alone (+/−2.3 cm).

US measurements were not possible (due to large or diffuse lesions, or acoustic shadowing on US images) in 14 patients (10.2 % of patients with MRI). Patients without US had significantly larger tumors at pathology (mean 5.3 vs 2.0 cm; *p* = 0.003); were more likely to be diagnosed with advanced (stage III/IV) disease (83.3 % vs 32.3 %; *p* = 0.001); were less likely to have received taxane-based NAC (38.5 % vs 74.0 %; *p* = 0.02); and were more likely to have undergone mastectomy (78.6 % vs 46.3 %; *p* = 0.02) than patients with US measurements. For the 14 patients *without* US, the MD between MRI and pathology was −1.5 cm (95 % CI: −3.1-0.1 cm) and the LOA were +/−6.0 cm (Table 2).

#### MRI versus mammography

For patients with pathologic residual tumor and measurements by MRI and mammography (*N* = 78), tumors with measurements of 0.0 cm by the tests typically measured ≤2.0 cm at pathology (Fig. 1c); however, the proportion of “missed” tumors within that range was higher for mammography (23.1 %) than MRI (10.3 %; *p* = 0.002). Mammography “missed” two tumors measuring >6.0 cm; one of those (measuring 11.0 cm) also measured 0.0 cm on MRI.

Pooled MDs showed a tendency for MRI to slightly overestimate pathologic tumor size (MD = 0.1 cm) with no evidence of heterogeneity (I^2^ = 0 %) (Table 2; Additional file 1: Appendix 11). No systematic bias was observed for mammography (MD = 0.0 cm), but moderate heterogeneity was present (I^2^ = 39 %). No evidence of a difference between MRI and mammographic measurements was observed (assuming a fixed effect, *p* = 0.59). Pooled LOA for mammography (+/−5.0 cm) were wider than for MRI (+/−4.1 cm) (Table 2); over- and underestimation were observed for both tests (Additional file 1: Appendices 5 and 7). Combining MRI and mammography by taking their mean did not improve the MD (0.1 cm) or LOA (+/−4.2 cm) over MRI alone.

Tumor measurements by mammography were not possible (due to dense breasts, tumor margins no longer being assessable, or tumor not being visible) for 25 patients (24.3 % of patients with MRI). Patients without mammography were significantly younger (mean 42 vs 47 years; *p* = 0.03) than patients with mammographic measurements. For those patients, the MD between MRI and pathology was 0.0 cm (95 % CI −0.7-0.7 cm) and the LOA were +/−3.5 cm (Table 2).

#### MRI versus clinical examination

For 107 patients with pathologic residual tumor and paired measurements by MRI and clinical examination, tumors “missed” by MRI measured ≤2.0 cm at pathology in all but one case (0.9 %), but 10 patients (9.3 %) with measurements of 0.0 cm by clinical examination had pathologic residual tumor >2.0 cm (*p* = 0.003). Both tests “missed” one tumor with a pathologic measurement of 11.0 cm (Fig. 1d).

Pooled MDs showed no systematic bias in MRI’s estimation of pathologic tumor size (MD = 0.0 cm) with no evidence of heterogeneity (I^2^ = 0 %) (Table 2; Additional file 1: Appendix 11). A relatively large tendency for underestimation by clinical examination (MD = −0.8 cm) was observed with moderate heterogeneity (Q = 4.65, df = 2, *p* = 0.1; I^2^ = 57 %); the pooled MD assuming a fixed effect was similar (MD = −0.7 cm). Pooled differences between MRI and clinical examination showed measurements by clinical examination to be significantly lower than MRI (assuming random effects, *p* = 0.006). Pooled LOA for clinical examination (+/−5.1 cm) were wider than for MRI (+/−4.2 cm) (Table 2); over- and underestimation were observed for both tests (Additional file 1: Appendices 5 and 8). Combining MRI and clinical examination by taking their mean did not substantially improve the MD (−0.2 cm) or LOA (+/− 4.1) over MRI alone.

Estimation of tumor size by clinical examination was not possible for three patients. In one patient each, MRI correctly estimated, underestimated (−0.1 cm) and overestimated (0.8 cm) pathologic tumor size.

#### MRI measurement by NAC agents and HER2 status

In 88 patients treated with non-taxane-based NAC from three studies [25, 29, 46], the pooled MD showed slight underestimation by MRI (−0.1 cm) with no evidence of heterogeneity (I^2^ = 0 %). Data from 63 patients treated with taxane-containing NAC in those studies showed a tendency for overestimation by MRI (MD = 0.2 cm) with no evidence of heterogeneity (I^2^ = 0 %) (Additional file 1: Appendix 12). Pooled LOA in patients treated with non-taxane-based NAC (+/−4.3 cm) were wider than for patients treated with taxanes (+/−2.8 cm). When three additional studies [13, 24, 38] using only taxane-containing NAC were included in pooled estimates (six studies, 152 patients in total), the MD did not change (0.2 cm; I^2^ = 0 %), but LOA were higher (+/−3.9 cm).

Pooled MDs from three studies [24, 29, 46] showed comparable overestimation by MRI in HER2- (MD = 0.2 cm; *N* = 97) and HER2+ patients (MD = 0.3 cm; *N* = 42), with no evidence of heterogeneity for either group (I^2^ = 0 %) (Additional file 1: Appendix 12). Pooled LOA were also similar (+/−4.3 cm for HER2- patients; +/− 4.2 cm for HER2+ patients).

### MRI when no residual tumor at pathology (pCR)

**Fig. 2:**
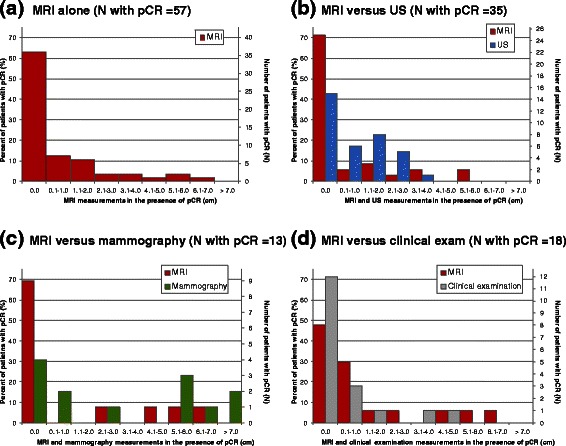
MRI measurements (cm) for: **a** all patients with pCR (*N* = 57); and compared with measurements by **b** US (*N* = 35), **c** mammography (N = 13), and **d** clinical examination (*N* = 18). Measurements of 0.0 cm denote correct identification of pCR. MRI = magnetic resonance imaging; pCR = pathologic complete response; US = ultrasound

For all studies combined, pCR was present in 57/300 patients (19.0 % [95 % CI: 14.7-23.9 %]). Study-specific rates of pCR ranged from 7.1-27.5 % (median = 19.1 %). MRI tumor measurements > 0.0 cm for patients with pCR are presented in Fig. 2a (measurements of 0.0 cm are also described, representing true identification of pCR by MRI). MRI measurements >0.0 cm ranged between 0.3-6.1 cm (median = 2.0 cm), and measured 0.1-1.0 cm for seven patients (12.3 %); 1.1-2.0 cm for six patients (10.5 %); 2.1-5.0 cm for five patients (8.8 %); and >5.0 cm for three patients (5.3 %).

#### MRI versus alternative tests in assessing pCR

Figure 2b–d present the distribution of MRI tumor measurements > 0.0 cm for patients with pCR compared with measurements by US (*N* = 35), mammography (*N* = 13, excluding five patients with MRI but no mammographic measurement), and clinical examination (*N* = 18). Large (>5.0 cm) measurement errors in the presence of pCR were more common by mammography (46.2 %) than MRI (15.4 %; *p* = 0.05); both large MRI measurements also measured >5.0 cm on mammography. The proportion of large MRI measurement errors was not significantly different from US or clinical examination.

For 5/18 patients (27.8 %) with no mammographic measurement (due to dense breasts or tumor margins not being assessable post-NAC), MRI measurements >0.0 cm occurred in three patients, ranging between 1.1–2.0 cm.

## Discussion

In the neoadjuvant setting, accurate measurement of residual malignancy may assist in guiding surgical management of breast cancer. While past research focussed on the accuracy of MRI to detect the absence of residual tumor (pCR) as a predictor of overall and disease-free survival [1], MRI measurements of tumor size have the potential to inform decisions about surgical extent (e.g. BCS versus mastectomy). Our IPD meta-analysis assessed the agreement between MRI and pathologic tumor measurements after NAC. Pooled MDs between MRI and pathology indicated that there was no systematic bias in MRI’s estimation of tumor size when residual tumor was present. Measurement variability for agreement was lower than estimated by our previous study-level analysis [14]; however, both over- and underestimation by MRI were observed, and LOA (+/−3.8 cm) show that substantial disagreement with pathology is possible. MRI measurement errors within that range may be of clinical importance in terms of their implications for the choice of treatment.

The IPD methodology used in this analysis allowed for measurement errors to be explored in greater detail than that permitted by study-level analyses [14]. Tumors “missed’ by MRI generally measured ≤2.0 cm at pathology; however, MRI measurements >5.0 cm occurred in a small proportion of cases where pCR was achieved. Although descriptive reporting of such overestimation was not standard across included studies, one of the three cases of MRI measurements >5 cm in the presence of pCR observed in this data set was attributed to the presence of extensive DCIS. Other possible causes include reactive inflammation, fibrosis or necrosis induced by NAC [13]. Description of cases of large overestimation in future studies would be valuable in guiding future research and practice. Assuming that surgeons consider the MRI-determined measurement when planning resection, such overestimation would lead to unnecessarily large excision. Although those patients are likely to benefit from improved disease-free and overall survival conferred by pCR [47], they are less likely to benefit from a reduction in surgical extent after NAC.

Comparisons of MRI and US *in the same patients* showed similar LOA, suggesting comparable performance by MRI and US when residual tumor is present (although substantial heterogeneity for US reflects its operator dependence [2]). However, contrary to our previous study-level analysis [14], a small bias towards underestimation of tumor size was found for US; clinical preference for either slight overestimation (MRI) or underestimation (US) of pathologic size should be considered in the choice of test. Furthermore, our analysis extends previous work by suggesting that considering the mean measurement of both tests may further improve tumor measurement. Given that studies may not have interpreted MRI blinded to US, this result is likely to underestimate the value of combining the tests. Clinicians adopting this testing strategy should be aware that the direction of MRI’s systematic bias was reversed (slight underestimation) when the tests were combined.

It is noteworthy that MRI did not estimate tumor size as accurately in patients for whom US measurement was not possible, with (on average) relatively large underestimation and wide LOA. Tumor characteristics are likely to have contributed to measurement being challenging for both tests. Patients without US had larger tumors (and consistent with this, were diagnosed with more advanced disease and were more likely to have undergone mastectomy), reflecting limitations in the US field of view [10]. The higher rate of non-taxane-based NAC in that group may also have contributed to the larger residual tumor size [48]. When planning resection, clinicians should note that although tumor measurement by MRI may be possible for such patients, the potential for size underestimation may lead to incomplete excision. This analysis is the first to consider those patients separately, and directly compare MRI and US when measurement by both tests can be undertaken. Our findings highlight the importance of study authors reporting MRI’s agreement with pathology separately for patients with and without alternative tests [14, 18].

In patients with measurements by both MRI and mammography, a systematic bias in estimating tumor size was found only for MRI (slight overestimation); the larger overestimation for mammography found in a previous analysis (which included fewer studies comparing mammography and MRI) [14] was not observed. However, the difference between test measurements was small, and mammography’s moderate heterogeneity, wider LOA, and tendency to “miss” smaller tumors (≤2.0 cm) indicate greater variability for agreement with pathology. Consequently, combining MRI and mammography did not improve tumor measurement compared with MRI alone. In addition, a tendency for large mammographic measurements in the presence of pCR suggests that mammography may lead to overly radical surgery when pCR is achieved. Mammographic tumor measurements were frequently not possible due to breast density, reflected in the younger age of those women [49]. These findings therefore suggest that MRI would be the preferred test in this setting.

Direct comparison of MRI and clinical examination showed no systematic bias in MRI’s measurement of residual tumor; relatively large underestimation, moderate heterogeneity and wider LOA for clinical examination were observed, suggesting greater variability for agreement with pathology. In addition, apart from one case, tumors with pathologic measurements of >2.0 cm were “missed” only by clinical examination, highlighting the potential for inadequate resection if surgical planning was based on clinical examination alone. While better overall agreement between MRI and pathology suggest that MRI is the more appropriate assessment method, it is possible that a combination of US and clinical examination may be superior to either test individually [50], but that testing strategy could not be explored in this analysis. The relative performance of test combinations should be considered in future studies.

Data from single studies have suggested that underestimation by MRI is common in HER2- patients [16] or those treated with taxane-containing regimens [17], but previous study-level meta-analyses were unable to further explore the effect of these variables. Similar effects were not observed in our IPD analysis. For patients with data available on HER2 status, MRI performed comparably regardless of tumor biology. Although that analysis was based on relatively few studies, the combined sample size is substantially larger than the previous study exploring the effect of this variable, and the studies that did not contribute data predate the routine testing of HER2. Furthermore, contrary to previous reports, a slight bias towards underestimation (and poorer overall agreement with pathology) was found in patients treated with non-taxane-based NAC. However, although more detailed analyses were attempted, statistical models were unstable and therefore the results presented are primarily descriptive. Further exploration of the effect of these characteristics on measurement accuracy is warranted in large primary studies, controlling for the effect other potentially important covariates.

Given that not all eligible studies contributed IPD to this meta-analysis, selection bias may have been introduced. Although studies in this analysis were similar in most respects to the broader population of eligible studies [14], a higher proportion of T3 tumors and stage III disease was apparent. Other differences suggest that included studies are more applicable to current practice (i.e. NAC with taxanes was more common), and less susceptible to changes in tumor dimensions between MRI and pathologic measurement (i.e. shorter interval between tests). Our IPD analysis also included a larger number of studies than the only previous (study-level) meta-analysis utilising appropriate statistical techniques to address this clinical question [14] (see Additional file 1: Appendix 1).

Although MDs and LOA are the most methodologically appropriate measures of agreement between MRI and pathology [15], there was no clear indication to consider either absolute or relative differences between the tests in our analysis. Plots of the data suggest that the absolute MDs reported here are likely to be most applicable to mid-sized tumors, but may differ for small or large residual cancers. However, analyses of absolute and relative differences were comparable, and therefore inferences about MRI and its performance compared to alternative tests are likely to be robust.

Due to pCR being achieved in a minority of patients (between 7.1 % and 27.5 % in the included studies), analyses of measurement errors in the presence of pCR are based on relatively small sample sizes and should therefore be interpreted cautiously. Furthermore, to standardise the definition of pCR across studies, this analysis considered the presence of invasive cancer only. This represents an advance in methods over previous analyses by reducing the potential for heterogeneity and improving the clinical applicability of pooled estimates. However, tests may differ in their ability to visualise DCIS or calcifications [11], and hence the accuracy of MRI and alternative tests to measure those outcomes may differ from our estimates. Our findings that alternative tests could not evaluate residual tumor in a proportion of patients should also be interpreted with awareness that corresponding data about non-evaluable tumors by MRI were unavailable.

## Conclusion

Our meta-analysis is the largest and most statistically appropriate evaluation of the agreement between MRI and pathologic residual tumor size post-NAC, and the only meta-analysis on this topic using IPD methodology. Our work suggests that there is no systematic bias in MRI’s measurement of residual invasive tumor, but that both over- and underestimation by MRI is possible, with LOA large enough to be of clinical importance. MRI’s performance was generally superior to that of US, mammography, and clinical examination, and in light of those findings, MRI may be considered the most appropriate test in this setting. However, large MRI measurements are possible in a small proportion of pCR cases, and patient characteristics that render tumors non-evaluable by US may contribute to inaccurate size measurements by MRI; those potential disadvantages should be considered in the choice of test. Furthermore, it is possible that a combination of US and clinical examination may be superior to those tests individually, and such a testing strategy has potential advantages over MRI in terms of lower cost and greater accessibility. Combinations of alternative tests, and their performance relative to MRI, should be explored in future studies.

## Additional file

Additional file 1: **Appendix 1.** Methodological comparison of IPD meta-analysis and previous study-level analysis of agreement between MRI and pathologic tumor measurements post-NAC. **Appendix 2.** PRISMA flowchart. **Appendix 3.** Research protocol and data collection template. **Appendix 4.** MRI technical characteristics of studies included in the IPD analysis. **Appendix 5.** Bland Altman Plots for MRI (absolute and log transformed values). **Appendix 6.** Bland Altman Plots for US (absolute and log transformed values). **Appendix 7.** Bland Altman Plots for mammography (absolute and log transformed values). **Appendix 8.** Bland Altman Plots for clinical examination (absolute and log transformed values). **Appendix 9.** Pooled relative differences (%) (fixed effect unless noted) and limits of agreement for studies and patients comparing the respective tests. **Appendix 10.** Forest plots of MRI and comparator tests (*relative* mean differences with pathology). **Appendix 11.** Forest plots of MRI and comparator tests (*absolute* mean differences with pathology). **Appendix 12.** Forest plots of MRI by chemotherapy agent and HER2 status (*absolute* mean differences with pathology). (DOC 796 kb)
