# Supplementary material for: Agreement between MRI and pathologic breast tumor size after neoadjuvant chemotherapy, and comparison with alternative tests: individual patient data meta-analysis
Source: BMC Cancer. 2015 Oct 8;15:662. doi: 10.1186/s12885-015-1664-4 (PMC4599727; doi:10.1186/s12885-015-1664-4)
Supplement: Additional file 1: — Appendix 1. Methodological comparison of IPD meta-analysis and previous study-level analysis of agreement between MRI and pathologic tumor measurements post-NAC. Appendix 2. PRISMA flowchart. Appendix 3. Research protocol and data collection template. Appendix 4. MRI technical characteristics of studies included in the IPD analysis. Appendix 5. Bland Altman Plots for MRI (absolute and log transformed values). Appendix 6. Bland Altman Plots for US (absolute and log transformed values). Appendix 7. Bland Altman Plots for mammography (absolute and log transformed values). Appendix 8. Bland Altman Plots for clinical examination (absolute and log transformed values). Appendix 9. Pooled relative differences (%) (fixed effect unless noted) and limits of agreement for studies and patients comparing the respective tests. Appendix 10. Forest plots of MRI and comparator tests (relative mean differences with pathology). Appendix 11. Forest plots of MRI and comparator tests (absolute mean differences with pathology). Appendix 12. Forest plots of MRI by chemotherapy agent and HER2 status (absolute mean differences with pathology). (DOC 796 kb) [file 12885_2015_1664_MOESM1_ESM.doc]

**Online Appendix 1: Methodological c**omparison of IPD meta-analysis and previous study-level analysis of agreement between MRI and pathologic tumor measurements post-NAC

|  | **IPD meta-analysis** | **Study-level meta-analysis** |
| --- | --- | --- |
| Number of included studies | 8 | 5 |
| Number of included patients | 300 | 264 |
| Estimates of systematic measurement bias (mean differences) and variability (limits of agreement) for MRI | Yes | Yes |
| Assessment of validity of assumptions underlying statistical analyses | Yes | No |
| Consideration of absolute vs. relative systematic bias | Yes | No |
| Estimates of bias when residual tumour truly present | Yes | No |
| Standardisation of reference standard (pathology) measurements | Yes | No |
| Detailed exploration of measurement errors | Yes | No |
| Comparison of results from appropriate and inappropriate analytic methods | No | Yes |
| Comparison of MRI and alternative tests | Yes | Yes |
| MRI, alternative tests and reference standard performed in same patients | Yes | No |
| Estimates of bias when studies performed tests in different patients | Yes | No |
| Comparison of test combinations | Yes | No |
| Investigation of factors proposed to modify MRI accuracy | Yes | No |

**Online Appendix 2: PRISMA flowchart**

Records identified through database searching
(n = 2,194)

Additional records identified through other sources
(n = 41)

Records after duplicates removed
(n = 2,108)

Records screened
(n = 2,108)

Full-text articles assessed for eligibility
(n = 159)

Records excluded, with reasons
(n = 1,949)

1 – wrong patients (n = 616)

2 – wrong test/not MRI (n = 188)

3 – wrong outcome (n = 126)

4 – wrong study type (n = 1,019)

Full-text articles excluded, with reasons
(n = 135)

1 – wrong patients (n = 5)

2 – wrong test/not MRI (n = 1)

3 – wrong outcome (n = 32)

4 – wrong study type (n = 45)

5 – not in English (n = 17)

6 – superseded publication (n = 3)

7 – MRI conducted during (not after)
NAC (n =9)

8 – No comparator (n = 23)

Studies invited to participate in IPD meta-analysis
(n = 24)

Studies excluded, with reasons
(n = 16)

1 – Did not respond (n = 11)

2 – Data unavailable or lost (n = 4)

3 – Declined to participate (n = 1)

Studies included in IPD meta-analysis:
8 studies

300 patients

**Online Appendix 3: Research protocol and data collection template**

**Estimation of tumour size by MRI and conventional assessment methods at the end of neoadjuvant chemotherapy (NAC): Individual patient data (IPD) meta-analysis**

**Research protocol**

**Luke Marinovich, Nehmat Houssami, Les Irwig, Petra Macaskill, Meagan Brennan**
Screening and Test Evaluation Program (STEP), Sydney Medical School, Australia

**Francesco Sardanelli**Dipartimento di Scienze Medico-Chirurgiche, Università Degli Studi di Milano, Italy

**Stefano Ciatto**
Breast Screening Program, Padua, Italy

**Gunter von Minckwitz**
German Breast Group, Neu-Isenburg, Germany

**Terry Mamounas**
Aultman Health Foundation, Canton, OH, United States

**1. Background**

Accurate estimation of the size of residual breast tumour after neoadjuvant chemotherapy (NAC) is essential to plan the extent of subsequent surgery (breast conservation vs mastectomy), and to minimise the need for re-operation. Tumour size assessment at the end of NAC has typically been undertaken by clinical examination and conventional imaging (mammography, ultrasound [US]), and more recently using magnetic resonance imaging (MRI).

Studies which compare MRI and other tests against pathologically-determined tumour size allow assessment of the extent to which MRI measurements agree with “actual” size, as well as whether estimation of tumour size by MRI is more accurate than other methods*.* Intraclass correlation coefficients (ICCs) may be used to assess agreement, however a systematic literature search has indicated that ICCs are seldom reported. Most studies report Pearson or Spearman correlation coefficients, which assess the association between the size measurements but not their agreement. The deficiencies of using Pearson or Spearman rank correlation coefficients, or simple measures such as the proportion of cases for which the tests and pathology “agree”, are described by Bland and Altman (1986). They also describe appropriate graphical and analytic methods which allow systematic biases in size measurement to be investigated. Application of these methods requires access to the individual tumour measurements.

Therefore, an individual patient data (IPD) meta-analysis is proposed to allow the relationships between pathologically-determined tumour size and measurements by MRI after NAC to be explored, and compared with any of the alternative conventional assessment methods (clinical examination, mammography and US).

**2. Benefits of IPD over study-level meta-analysis**

Access to data at the level of individual patients/tumours will allow:

1. Standardisation of analyses – statistical analyses reported across studies are highly variable, rendering study-level comparisons and data pooling problematic;
2. Appropriate statistical analyses (such as those described in 3.3) and graphical presentations of the data – commonly presented analyses assess the *association* between tumour measurements; re-analysis to consider *agreement* cannot be undertaken on study-level data;
3. Standardisation of data on important covariates such as patient age and type of NAC, and investigation of their effect on agreement.

**2. Objectives**

To determine the accuracy of MRI (relative to clinical examination, mammography and US) to estimate residual tumour extent after NAC (prior to surgery).

The aims are to use IPD from published reports to:

1. Estimate pooled ICCs for MRI (and comparator tests) versus pathologically determined tumour size, using important covariates to investigate heterogeneity between studies.
2. Estimate pooled mean differences (as an estimate of systematic bias) in the measurement of tumour size by MRI (and comparator tests) and pathology, as well as random measurement variability in size measurement by MRI (and comparator tests), using important covariates to investigate heterogeneity between studies.

**3. Methods**

*3.1 Identification and inclusion of studies*

Studies reporting a comparison of MRI with clinical assessment, mammography or US, in which size measurements are validated against tumour size from pathology, were identified through a systematic review of the biomedical literature.

*3.2 Data items*

A table with the requested data items is presented in the attachment (*Example Data Table*).

Minimum data items requested for each patient (or each tumour, where measurements of multiple tumours have been undertaken):

- Tumour size measurement on MRI (mm)
- Tumour size measurement on mammography, and/or US, and/or clinical examination (mm)
- Tumour size measurement on pathological examination (mm)
- Indicator of whether measurement is based on a single tumour or a composite of multiple tumours
- Age (years)

Additional data items requested, if available:

- Additional tumour size measurements by MRI, pathology and comparator tests, if performed (e.g. perpendicular diameter) (mm)
- Clinical T stage or clinical stage (pre-NAC)
- Chemotherapy agent(s)
- Time between MRI and surgery (days)
- Type of surgery performed (BCS, mastectomy)
- ER, PR and HER2 status

Confirmation of how clinical examination, imaging and pathological size measurements were undertaken will also be requested (see attached *Example Data Table*).

*3.3 Statistical analysis*

3.3.1 Core analyses

The ICC and its variance will be computed for each study. Individual ICCs will be pooled using the variance estimate for each study. Age and the additional variables collected will be included as study-level covariates.

Bland-Altman plots will be generated for each study (Bland & Altman, 1986). If the difference in size measurements between the test(s) and pathology do not depend on underlying mean size, mean differences (as estimates of systematic bias) and their variances will be calculated for each study, and pooled. Where differences in size measurement are proportional to mean size, log transformation will be applied (representing proportional systematic bias after back-transformation). Meta-regression using study-level covariates will be undertaken.

If feasible, a multilevel model will be fit that will include random effects for studies, tumours within studies, and the pairing of measurements (test, pathology) within tumours. Tumour and study level covariates will also be considered for inclusion in this model.

3.3.2 Data checking

Statistics reported in the original paper will be recalculated, and discrepancies will be clarified with study authors (to ensure, for example, that analyses are conducted on the same cases and tumour measurements).

3.3.3 Additional analyses

IPD will be used to investigate the sensitivity and specificity of MRI and other tests in determining pathologic complete response at the end of NAC, where size measurements of zero are taken to indicate absence of tumour on tests and pathology.

**4. References**

Bland, JM and Altman, DG (1986) Statistical methods for assessing agreement between two methods of clinical measurement. *Lancet*, 327(8476), 307-310.

**Example table of requested data: *[study author, year]* *(reference number)***

Double-click on tables to open as an Excel sheet. Please complete **all fields in black**, and **fields in red where data is available**.

Study-level data items continue on pages 2-5.

**Classification of tumour size measurements**

| **1. For the data provided on tumour size measured by** **MRI, how was tumour size defined? *(Please complete sections A and B)*** | | | | |
| --- | --- | --- | --- | --- |
| **Section 1A:** | | | | |
| Please mark *one* option (X) | | | **For subjects with a single tumour only, including those with multifocal tumours where only the main (largest) tumour is measured:** | |
|  |  |  | | Maximum unidimensional diameter of single tumour |
|  |  |  | | Maximum and perpendicular (bidimensional) diameters of single tumour, reported separately |
|  |  |  | | *Sum* of two dimensions of single tumour |
|  |  |  | | *Product* of two dimensions of single tumour |
|  |  |  | | *Mean* of two dimensions of single tumour |
|  |  |  | | Tumour volume |
|  |  |  | | Other – please describe: |
|  | | | | |
| **Section 1B:** | | | | |
| Please mark *one* option (X) | | | **For subjects with multifocal tumours, where multiple tumours are combined into a single composite measurement:** | |
|  |  |  | | Sum of measurements of individual tumours |
|  |  |  | | Maximum diameter of region occupied by individual tumours, including intermingled healthy tissue |
|  |  |  | | Other – please describe: |
|  |  |  | | Not applicable |
|  | | | | |

| **2. For the data provided on tumour size measured by clinical examination, how was tumour size defined? *(Please complete sections A and B)*** | | | | |
| --- | --- | --- | --- | --- |
| **Section 2A:** | | | | |
| Please mark *one* option (X) | | | **For subjects with a single tumour only, including those with multifocal tumours where only the main (largest) tumour is measured:** | |
|  |  |  | | Maximum unidimensional diameter of single tumour |
|  |  |  | | Maximum and perpendicular (bidimensional) diameters of single tumour, reported separately |
|  |  |  | | *Sum* of two dimensions of single tumour |
|  |  |  | | *Product* of two dimensions of single tumour |
|  |  |  | | *Mean* of two dimensions of single tumour |
|  |  |  | | Tumour volume |
|  |  |  | | Other – please describe: |
|  | | | | |
| **Section 2B:** | | | | |
| Please mark *one* option (X) | | | **For subjects with multifocal tumours, where multiple tumours are combined into a single composite measurement:** | |
|  |  |  | | Sum of measurements of individual tumours |
|  |  |  | | Maximum diameter of region occupied by individual tumours, including intermingled healthy tissue |
|  |  |  | | Other – please describe: |
|  |  |  | | Not applicable |
|  | | | | |

| **3. For the data provided on tumour size measured by** **mammography, how was tumour size defined? *(Please complete sections A and B)*** | | | | |
| --- | --- | --- | --- | --- |
| **Section 3A:** | | | | |
| Please mark *one* option (X) | | | **For subjects with a single tumour only, including those with multifocal tumours where only the main (largest) tumour is measured:** | |
|  |  |  | | Maximum unidimensional diameter of single tumour |
|  |  |  | | Maximum and perpendicular (bidimensional) diameters of single tumour, reported separately |
|  |  |  | | *Sum* of two dimensions of single tumour |
|  |  |  | | *Product* of two dimensions of single tumour |
|  |  |  | | *Mean* of two dimensions of single tumour |
|  |  |  | | Tumour volume |
|  |  |  | | Other – please describe: |
|  | | | | |
| **Section 3B:** | | | | |
| Please mark *one* option (X) | | | **For subjects with multifocal tumours, where multiple tumours are combined into a single composite measurement:** | |
|  |  |  | | Sum of measurements of individual tumours |
|  |  |  | | Maximum diameter of region occupied by individual tumours, including intermingled healthy tissue |
|  |  |  | | Other – please describe: |
|  |  |  | | Not applicable |
|  | | | | |

| **4. For the data provided on tumour size measured by** **pathology, how was tumour size defined? *(Please complete sections A, B and C)*** | | | | |
| --- | --- | --- | --- | --- |
| **Section 4A:** | | | | |
| Please mark *one* option (X) | | | **For subjects with a single tumour only, including those with multifocal tumours where only the main (largest) tumour is measured:** | |
|  |  |  | | Maximum unidimensional diameter of single tumour |
|  |  |  | | Maximum and perpendicular (bidimensional) diameters of single tumour, reported separately |
|  |  |  | | *Sum* of two dimensions of single tumour |
|  |  |  | | *Product* of two dimensions of single tumour |
|  |  |  | | *Mean* of two dimensions of single tumour |
|  |  |  | | Tumour volume |
|  |  |  | | Other – please describe: |
|  | | | | |
| **Section 4B:** | | | | |
| Please mark *one* option (X) | | | **For subjects with multifocal tumours, where multiple tumours are combined into a single composite measurement:** | |
|  |  |  | | Sum of measurements of individual tumours |
|  |  |  | | Maximum diameter of region occupied by individual tumours, including intermingled healthy tissue |
|  |  |  | | Other – please describe: |
|  |  |  | | Not applicable |
|  | | | | |

| **Section 4C:** | | | | |
| --- | --- | --- | --- | --- |
| Please mark *one* option (X) | | | **Were ductal carcinoma *in situ* (DCIS) components included in pathologic size measurement?** | |
|  |  |  | | Yes |
|  |  |  | | No |
|  | | | | |

**Classification of MRI parameters**

| **5. For MRI, *please* *complete sections A, B and C*:** | | | | |
| --- | --- | --- | --- | --- |
| **Section 5A:** | | | | |
| Please mark *one* option (X) | | | **On which images were the diameter(s) measured?** | |
|  |  |  | | Subtracted |
|  |  |  | | Fat-saturated |
|  |  |  | | Other – please describe: |
|  | | | | |
| **Section 5B:** | | | | |
| Please mark *one* option (X) | | | **In which plane were the diameter(s) measured?** | |
|  |  |  | | Axial |
|  |  |  | | Coronal |
|  |  |  | | Sagittal |
|  |  |  | | Oblique multiplanar reconstructions |
|  |  |  | | Maximum intensity projections |
|  | | | | |
| **Section 5C:** | | | | |
| Please mark *one* option (X) | | | **On which dynamic phase** **were the diameter(s) measured?** | |
|  |  |  | | Within 60 seconds after contrast injection |
|  |  |  | | From 61 to 120 seconds after contrast injection |
|  |  |  | | From 121 to 180 seconds after contrast injection |
|  |  |  | | From 181 to 240 seconds after contrast injection |
|  |  |  | | From 241 to 300 seconds after contrast injection |
|  |  |  | | Beyond 300 seconds after contrast injection |
|  |  |  | | Measures were taken in different phases, on a case-by-case basis, according to the evaluation of the reader(s) |
|  |  |  | |
|  | | | | |

**Online Appendix 4: MRI technical characteristics of studies included in the IPD analysis (online only)**

| **Variable** | **Number of studies (%)** | **Number of patients (%)** |
| --- | --- | --- |
| ***MRI*** |  |  |
| **Type** |  |  |
| CE | 0 (0.0) | 0 (0.0) |
| DCE | 7 (87.5) | 260 (86.7) |
| Unknown | 1 (12.5) | 40 (13.3) |
| **Field (T)** |  |  |
| 1.5 | 6 (75.0) | 247 (82.3) |
| <1.5 | 1 (12.5) | 13 (4.3) |
| Unknown | 1 (12.5) | 40 (13.3) |
| **Contrast type** |  |  |
| Gadopentetate dimeglumine | 5 (62.5) | 153 (51.0) |
| Gadolinium other/not specified | 2 (25.0) | 107 (35.7) |
| Unknown | 1 (12.5) | 40 (13.3) |
| **Contrast dose (mmol/kg)** |  |  |
| 0.1 mmol/kg | 6 (75.0) | 228 (76.0) |
| >0.1 mmol/kg | 1 (12.5) | 32 (10.7) |
| Unknown | 1 (12.5) | 40 (13.3) |

*Abbreviations*: CE = contrast enhanced; DCE = dynamic contrast enhanced; MRI = magnetic resonance imaging; T = tesla.

**Online Appendix 5: Bland Altman Plots for MRI (absolute and log transformed values)**

Figure 3: Bland-Altman plots and scatterplots for study-specific tumour size measurements by MRI and pathology. Black dots are true positives (i.e. tumour present on MRI when tumour is present on pathology); red dots are false negatives (i.e. MRI measurements of zero when tumour is present on pathology); green circles are false positives (i.e. tumour present on MRI when no tumour is present on pathology); blue circles are true negatives (i.e. no tumour present on MRI when no tumour is present on pathology). MRI false positives and true negatives are excluded from the calculation of mean differences and 95% limits of agreement.

|  |  |
| --- | --- |
|  |  |
|  |  |
|  |  |
|  |  |
|  |  |
|  |  |
|  |  |

**Figure 4: Bland-Altman plots (showing mean differences and 95% limits of agreement) and scatterplots (showing the line of identity) for study-specific tumour *log transformed*** size measurements by MRI and pathology. Black dots are true positives (i.e. tumour present on MRI when tumour is present on pathology); red dots are false negatives (i.e. MRI measurements of zero when tumour is present on pathology); green circles are false positives (i.e. tumour present on MRI when no tumour is present on pathology); blue circles are true negatives (i.e. no tumour present on MRI when no tumour is present on pathology). MRI false positives and true negatives are excluded from the calculation of mean differences and 95% limits of agreement.

|  |  |
| --- | --- |
|  |  |
|  |  |
|  |  |
|  |  |
|  |  |
|  |  |
|  |  |

**Online Appendix 6: Bland Altman Plots for US (absolute and log transformed values)**

Figure 5: Bland-Altman plots (showing mean differences and 95% limits of agreement) and scatterplots (showing the line of identity) for study-specific tumour size measurements by US and pathology. Black dots are true positives (i.e. tumour present on US when tumour is present on pathology); red dots are false negatives (i.e. US measurements of zero when tumour is present on pathology); green circles are false positives (i.e. tumour present on US when no tumour is present on pathology); blue circles are true negatives (i.e. no tumour present on US when no tumour is present on pathology). US false positives and true negatives are excluded from the calculation of mean differences and 95% limits of agreement.

|  |  |
| --- | --- |
|  |  |
|  |  |
|  |  |
|  |  |

**Figure 6: Bland-Altman plots (showing mean differences and 95% limits of agreement) and scatterplots (showing the line of identity) for study-specific tumour *log transformed*** size measurements by US and pathology. Black dots are true positives (i.e. tumour present on US when tumour is present on pathology); red dots are false negatives (i.e. US measurements of zero when tumour is present on pathology); green circles are false positives (i.e. tumour present on US when no tumour is present on pathology); blue circles are true negatives (i.e. no tumour present on US when no tumour is present on pathology). US false positives and true negatives are excluded from the calculation of mean differences and 95% limits of agreement.

|  |  |
| --- | --- |
|  |  |
|  |  |
|  |  |
|  |  |

**Online Appendix 7: Bland Altman Plots for mammography (absolute and log transformed values)**

Figure 7: Bland-Altman plots (showing mean differences and 95% limits of agreement) and scatterplots (showing the line of identity) for study-specific tumour size measurements by mammography and pathology. Black dots are true positives (i.e. tumour present on mammography when tumour is present on pathology); red dots are false negatives (i.e. mammography measurements of zero when tumour is present on pathology); green circles are false positives (i.e. tumour present on mammography when no tumour is present on pathology); blue circles are true negatives (i.e. no tumour present on mammography when no tumour is present on pathology). False positives and true negatives on mammography are excluded from the calculation of mean differences and 95% limits of agreement.

|  |  |
| --- | --- |
|  |  |
|  |  |
|  |  |

**Figure 8: Bland-Altman plots (showing mean differences and 95% limits of agreement) and scatterplots (showing the line of identity) for study-specific tumour *log transformed*** size measurements by mammography and pathology. Black dots are true positives (i.e. tumour present on mammography when tumour is present on pathology); red dots are false negatives (i.e. mammography measurements of zero when tumour is present on pathology); green circles are false positives (i.e. tumour present on mammography when no tumour is present on pathology); blue circles are true negatives (i.e. no tumour present on mammography when no tumour is present on pathology). False positives and true negatives on mammography are excluded from the calculation of mean differences and 95% limits of agreement.

|  |  |
| --- | --- |
|  |  |
|  |  |
|  |  |

**Online Appendix 8: Bland Altman Plots for clinical examination (absolute and log transformed values)**

**Figure 9: Bland-Altman plots (showing mean differences and 95% limits of agreement) and scatterplots (showing the line of identity) for study-specific tumour size measurements by clinical examination and pathology. Black dots are true positives (i.e. tumour present on clinical examination when tumour is present on pathology); red dots are false negatives (i.e. clinical examination measurements of zero when tumour is present on pathology); green circles are false positives (i.e. tumour present on clinical examination when no tumour is present on pathology); blue circles are true negatives (i.e. no tumour present on clinical examination** when no tumour is present on pathology). False positives and true negatives on clinical examination are excluded from the calculation of mean differences and 95% limits of agreement.

|  |  |
| --- | --- |
|  |  |
|  |  |

**Figure 10: Bland-Altman plots (showing mean differences and 95% limits of agreement) and scatterplots (showing the line of identity) for study-specific tumour *log transformed* size measurements by clinical examination and pathology. Black dots are true positives (i.e. tumour present on clinical examination when tumour is present on pathology); red dots are false negatives (i.e. clinical examination measurements of zero when tumour is present on pathology); green circles are false positives (i.e. tumour present on clinical examination when no tumour is present on pathology); blue circles are true negatives (i.e. no tumour present on clinical examination** when no tumour is present on pathology). False positives and true negatives on clinical examination are excluded from the calculation of mean differences and 95% limits of agreement.

|  |  |
| --- | --- |
|  |  |
|  |  |

**Online Appendix 9:** Pooled relative differences (%) (fixed effect unless noted) and limits of agreement for studies and patients comparing the respective tests

|  | **N (studies)** | **N (patients)** | **Pooled MD (%) (95% CI)** | **I^2^** | **LOA (%)** |
| --- | --- | --- | --- | --- | --- |
| **All studies and patients** |  |  |  |  |  |
| MRI vs pathology | 8 | 243 | 5 (-3, 14) | 0% | -84, 597 |
|  |  |  |  |  |  |
| **Studies of MRI vs US** |  |  |  |  |  |
| MRI vs pathology | 5 | 123 | 3 (-10, 17) | 0% | -87, 691 |
| US vs pathology^*^ | 5 | 123 | -14 (-36, 15) | 76% | -84, 398 |
| MRI *and* US (mean) vs pathology^*^ | 5 | 123 | 1 (-16, 21) | 57% | -81, 516 |
| MRI vs US^*^ | 5 | 123 | 21 (-10, 63) | 77% | NA |
| *MRI vs pathology (patients without US)^**^* | *3* | *14* | *-23 (-49, 16)* | *NA* | *-81, 214* |
|  |  |  |  |  |  |
| **Studies of MRI vs mammography** |  |  |  |  |  |
| MRI vs pathology | 4 | 78 | 5 (-9, 20) | 0% | -86, 675 |
| Mammography vs pathology | 4 | 78 | -20 (-45, 16) | 65% | -92, 1059 |
| MRI *and* mammography (mean) vs pathology | 4 | 78 | 6 (-8, 22) | 9% | -85, 770 |
| MRI vs mammography | 4 | 78 | 24 (-6, 64) | 52% | NA |
| *MRI vs pathology (patients without mammography)^**^* | *3* | *25* | *-4 (-26, 24)* | *NA* | *-72, 228* |
|  |  |  |  |  |  |
| **Studies of MRI vs clinical examination** |  |  |  |  |  |
| MRI vs pathology | 3 | 107 | 6 (-4, 18) | 2% | -79, 428 |
| Clinical exam vs pathology^*^ | 3 | 107 | -48 (-61, -30)^†^ | 32% | -97, 977 |
| MRI *and* clinical examination (mean) vs pathology | 3 | 107 | -4 (-15, 9) | 9% | -83, 559 |
| MRI vs clinical examination^*^ | 3 | 107 | 97 (52, 156)^†^ | 0% | NA |
| *MRI vs pathology (patients without clinical examination) ^**^* | *2* | *3* | *NA^‡^* | *NA^‡^* | *NA^‡^* |

^*^ Random effects
^**^ Patients without comparator test combined as a single data set; pooled meta-analysis not undertaken*.*^†^ *p*<0.01
^‡^ Not calculated due to small number of patients.
*Abbreviations*: CI = confidence interval; LOA = limits of agreement; MD = mean difference; MRI = magnetic resonance imaging; NA = not applicable; US = ultrasound.

**Online Appendix 10: Forest plots of MRI and comparator tests (*relative* mean differences with pathology)**

**Figure 11: Forest plot of relative mean difference between MRI and pathologic size (all studies, ordered by increasing MD)**

**Figure 12: Forest plots of relative mean difference between MRI or US and pathologic size (comparative studies, ordered by increasing MD for MRI)**

**Figure 13: Forest plots of relative mean difference between MRI or mammography and pathologic size (comparative studies, ordered by increasing MD for MRI)**

**Figure 14: Forest plots of relative mean difference between MRI or clinical examination and pathologic size (comparative studies, ordered by increasing MD for MRI)**

**Online Appendix 11: Forest plots of MRI and comparator tests (*absolute* mean differences with pathology)**

**Figure 15: Forest plot of absolute mean difference (MD) between MRI and pathologic size (all studies, ordered by increasing MD)**

**Figure 16: Forest plots of absolute mean difference between MRI or US and pathologic size (comparative studies, ordered by increasing MD for MRI)**

**Figure 17: Forest plots of absolute mean difference between MRI or mammography and pathologic size (comparative studies, ordered by increasing MD for MRI)**

**Figure 18: Forest plots of absolute mean difference between MRI or clinical examination and pathologic size (comparative studies, ordered by increasing MD for MRI)**

**Online Appendix 12: Forest plots of MRI by chemotherapy agent and HER2 status (*absolute* mean differences with pathology)**

**Figure 19: Forest plot of absolute mean difference (MD) between MRI and pathologic size in patients treated with non-taxane-based NAC (ordered by increasing MD)**

**Figure 20: Forest plot of absolute mean difference (MD) between MRI and pathologic size in patients treated with taxane-based NAC (ordered by increasing MD)**

**Figure 21: Forest plot of absolute mean difference (MD) between MRI and pathologic size in patients treated with HER2- tumors (ordered by increasing MD)**

**Figure 22: Forest plot of absolute mean difference (MD) between MRI and pathologic size in patients treated with HER2+ tumors (ordered by increasing MD)**
